# *Mustela strandi* (Mustelidae, Carnivora) from the Early Pleistocene of Crimea

**DOI:** 10.1134/S0012496623700680

**Published:** 2023-09-12

**Authors:** D. O. Gimranov, Q. Jiangzuo, A. V. Lavrov, A. V. Lopatin

**Affiliations:** 1 Institute of Plant and Animal Ecology, Ural Branch, Russian Academy of Sciences, 620144 Yekaterinburg, Russia; 2 Key Laboratory of Orogenic Belts and Crustal Evolution, School of Earth and Space Sciences, Peking University, 100871 Beijing, China; 3 Key Laboratory of Vertebrate Evolution and Human Origins of Chinese Academy of Sciences, Institute of Vertebrate Paleontology and Paleoanthropology, Chinese Academy of Sciences, 100044 Beijing, China; 4 Borissiak Paleontological Institute, Russian Academy of Sciences, 117647 Moscow, Russia

**Keywords:** *Mustela strandi*, mandible, Late Villafranchian, Taurida cave, Crimea

## Abstract

The dentary of *Mustela strandi* Kormos, 1934 is described from the Lower Pleistocene deposits (Late Villafranchian, 1.8–1.5 Ma) of the Taurida cave in Crimea. It is the first finding of *M. strandi* in Russia. This extinct mustelid species is rarely found in the Lower and Middle Pleistocene of Central Europe.

Small mustelids of the genus *Mustela* emerge in the Early Pliocene in the fossil record, and their finds throughout the Pliocene are extremely scarce. The most ancient species *Mustela plioerminea* Stach, 1959 and *M. pliocaenica* Stach, 1959 have been described from the localities Węże 1 (Poland, 3.6–3.2 Ma) and Wölfersheim (Germany, 3.6–3.5 Ma) [1, 2]. Two large lineages, stoats and weasels, diverged within the genus in the Pliocene; molecular genetic data support this divergence time [3].

Members of the genus *Mustela* increased in number and spread to Asia in the first half of the Pleistocene. The genus seems to reach its maximum species diversity in the Late Pleistocene and includes 17 or 18 species in the modern fauna [4].

*Mustela palerminea* (Petenyi, 1864), *M. praenivalis* Kormos, 1934, *M. strandi* Kormos, 1934, *M. putorius stromeri* Kormos, 1934, *M.* cf*. eversmanii* (Lesson, 1827), and *M. nivalis* Linnaeus, 1766 have been described from the Lower Pleistocene of Europe [5–8]. In Asia, *M.* cf. *sibirica* Pallas, 1773 [9] and *Mustela* sp. [10] were recorded. The species *M. palerminea* and *M. praenivalis* were the most widespread and abundant in the Early Pleistocene; their fossils have been found in many European localities [11–13]. We have recently discovered *M. palerminea* in Crimea [14].

The extremely rare European species *M. strandi* is poorly known. The species has first been found in the Hungarian locality Brasso (Middle Pleistocene) and initially described as *Putorius* (*Arctogale*) sp. [15]. The form has later been recognized as a separate species, *M. strandi* Kormos, 1934 [5]. More recently, *M. strandi* has been described from other five localities in Central Europe. In the Early Pleistocene, *M. strandi* inhabited the territories of Germany (Schernfeld, 1.9–1.6 Ma) and Poland (Żabia Cave, 1.7–1.5 Ma) [13]. Fossils of *M. strandi* dating to the boundary between the Early and Middle Pleistocene have been found in the same countries (Sackdilling Cave, Germany, 0.9–0.7 Ma; Kozi Grzbiet, Poland, 0.8–0.7 Ma) [13]. The find of *M. strandi* from the Południowa Cave (Kitzelhöhle) in southwestern Poland is dated to the middle part of the Middle Pleistocene [16].

Here we consider a *M. strandi* find from the Lower Pleistocene of the Taurida cave, Crimea (Belogorsk District, Zuya village). Based on the vertebrate faunal composition, the bone-bearing deposits of the Taurida cave is dated to the Early Pleistocene (Late Villafranchian, Psekupsian Faunal Assemblage, approximately 1.8–1.5 Ma) [17]. Mammals described from the locality include numerous large carnivores of the families Ursidae, Canidae, Felidae, and Hyaenidae and rare small Mustelidae [14].

We additionally examined the *M. strandi* holotype stored at the Geological Museum of Budapest (Hungary). Comparisons were performed with collections of the extant species *M. erminea* Linnaeus, 1758; *M. nivalis* Linnaeus, 1766; *M. eversmanii* (Lesson, 1827); *M. putorius* Linnaeus, 1758; *M. sibirica* Pallas, 1773; *M. altaica* Pallas, 1811; *M. lutreola* Linnaeus, 1761; and *Neogale vison* (Schreber, 1777); the collections are housed in the Institute of Plant and Animal Ecology, Ural Branch of the Russian Academy of Sciences (IPAE, Yekaterinburg), the Zoological Museum of Moscow State University, and the Zoological Institute of the Russian Academy of Sciences (St. Petersburg).

The specimen from the Taurida cave is a left dentary (specimen IPAE, no. 727/2263, collected in 2021). Measurements were performed with a caliper accurate to 0.01 mm. Data were visualized in R 4.1 [18], using the ggplot2 package [19].

The left mandibular ramus (specimen IPAE, no. 727/2263) includes unworn p4–m2. The incisor part of the mandible, the canine, and the symphysis are lost; the mandible is broken at the p3 level. The tip of the coronoid process is missing; major parts of the articular and angular processes are also missing (Fig. 1). The mandible is rather massive. The masseteric fossa (fossa masseterica) is oval and moderately deep; its anterior margin goes beyond the level of the posterior margin of m1. The posterior margin of the coronoid process (proc. coronoideus) is subvertical. The base of the angular process (proc. angularis) is massive. The articular process (proc. articularis) is positioned somewhat lower than the alveolar margin of the mandible. The edge of the insertion area of the medial part of the temporalis muscle (musculus temporalis pars medialis) on the coronoid process is well distinct and embossed. Its anterior part is ventral to the posterior margin of m2, and its ventral part is at the level of the middle of the articular process. A well-distinct relief and a low position of the edge of the attachment area on the mandible suggest strong specialized development of the temporalis muscle.

**Fig. 1.**
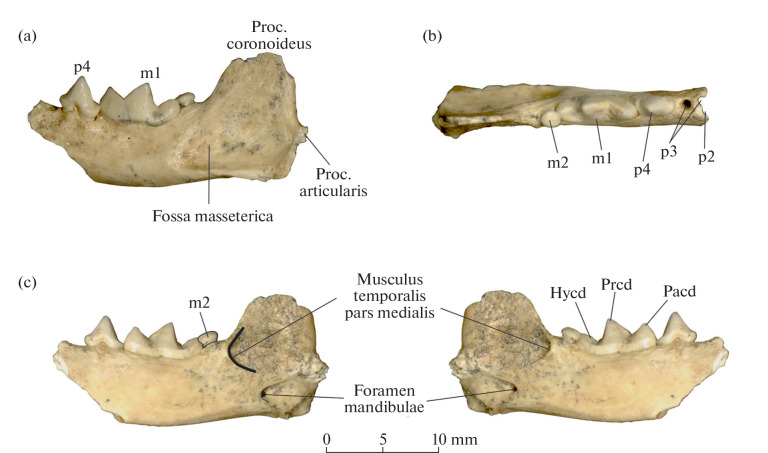
*Mustela strandi* Kormos, 1934, specimen IPAE, no. 727/2263, left dentary with p4–m2: (a) buccal view, (b) occlusal view, and (c) lingual view (with mirror image drawing); Crimea, Taurida cave; Lower Pleistocene. Designations: Hycd, hypoconid; Pacd, paraconid; Prcd, protoconid.

Because only a posterior part of the p2 socket is preserved, it is unclear whether p2 is single or double rooted. As judged from the alveoli, p3 is double rooted and is larger than p2, but smaller than p4. The posterior alveolus of p3 is slightly larger than the anterior one.

The ultimate premolar p4 is a rather large and massive tooth. The crown is somewhat asymmetric and slightly widens in its posterior part. The main cusp is conical and has a well-developed distal crest. There is no distinct anterior cingulid; the posterior cingulid without cusps. The posterior part of the p4 crown slightly overlaps the m1 paraconid on the buccal side. The apex of the main cusp of p4 is higher than the paraconid and protoconid of m1 (Fig. 1a). The enamel surface of p4 and m1 is smooth and lacks distinct crenulation.

The crown of the carnassial tooth m1 is large, extended longitudinally, and lacks projections in the middle part and a narrowing in the anterior part of the talonid. The lingual cingulid is nearly complete and is interrupted only in a small area of the middle part; the buccal cingulid is not developed. The paraconid is lower than the protoconid in m1 (Fig. 1a); the carnassial notch is deep and wide; the metaconid crest is absent. The talonid is of a shearing type, lacking a talonid basin. The talonid is elongated and nearly equal in length to the paraconid. The hypoconid is enlarged, has the appearance of a shearing crest, and occurs on the longitudinal axis of the tooth. The posterior cingulid cuspule is absent from the talonid. An additional root is not developed between the anterior and posterior roots.

The ultimate molar m2 is reduced, but still rather large and is displaced in the lingual direction relative to the longitudinal axis of m1 (Fig. 1b). The occlusal surface of m2 is located higher than the m1 talonid level (Fig. 1c). The m2 crown is a slightly elongated oval in shape. The flattened occlusal surface has a low narrow crest, which extends longitudinally.

Measurements of the dentary and teeth of specimen IPAE, no. 727/2263 are summarized in Table 1.

**Table 1.** Dentary measurements (mm) of *Mustela strandi* Kormos, 1934 from the Pleistocene of Europe (*n*, number of specimens)

Measurement	Taurida cave, Crimea (IPAE, no. 727/2263)	Kozi Grzbiet*, Poland			Brasso, Hungary
		range	mean	*n*	
Length of coronoid process base	9.03	–	–	–	–
Depth behind p4	5.56	–	–	–	–
Depth behind m1	6.51	5.5–6.3	5.7	3	–
Thickness under p4	3.0	–	–	–	–
Thickness under m1	2.96	2.8–3.4	3.0	3	–
Length of p3–m2	14.47	–	–	–	–
Length of p4–m1	10.27	–	–	–	–
Length of p4–m2	11.36	–	–	–	–
Length of m1–m2	7.98	–	–	–	–
Length of p4	3.62	3.3–3.7	3.5	4	3.4
Width of p4	1.97	1.4–1.9	1.6	5	1.8
Length of m1	6.8	6.2–6.7	6.5	4	6.65
Length of m1 trigonid	5.18	–	–	–	–
Width of m1 trigonid	2.48	2.2–2.7	2.4	4	2.35
Width of m1 talonid	2.07	1.5–1.8	1.7	4	1.6
Length of m2	1.55	1.6–2.0	1.8	4	1.5
Width of m2	1.45	1.5–1.9	1.7	4	1.4

* Dimensions are as in [5, 6].

The carnassial tooth m1 of specimen IPAE, no. 727/2263 is similar in length and width to those of *M. strandi* from the Middle Pleistocene locality Brasso in Hungary and the Early Pleistocene (terminal Early Pleistocene) locality Kozi Grzbiet in Poland (Fig. 2). It should be noted that specimen IPAE, no. 727/2263 has larger dentary measurements (the depth of the mandibular body behind m1, the length and width of p4, the length of m1, and the width of the m1 talonid) as compared with the specimens from Kozi Grzbiet and Brasso (Table 1). As is seen from Fig. 2, the m1 dimensions are substantially smaller in *M. palerminea* and greater in *M.* cf. *sibirica* from China, *M. putorius stromeri* and *M.* cf*. eversmanii* from Europe as compared with the m1 measurements of *M. strandi.*

**Fig. 2.**
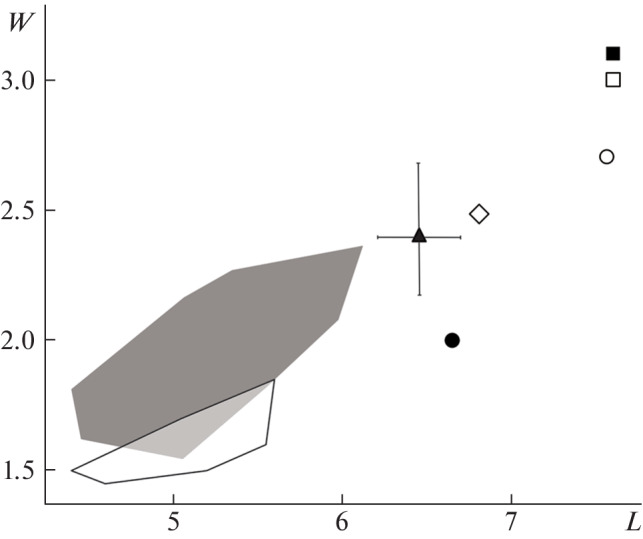
Measurements of m1 in *Mustela* representatives from the Pleistocene of Eurasia. Designations: L, length of m1; W, greatest width of m1; black square, *M. putorius stromeri* Kormos, 1934 [5]; white square, *M.* cf. *eversmanii* (Lesson, 1827) [7]; white circle, *M*. cf. *sibirica* Pallas, 1773 [9]; black triangle (with limits of variability) [6] and black circle [5], *M. strandi* Kormos, 1934; white diamond, specimen IPAE, no. 727/2263; gray [13] and white areas [13], *M. palerminea* (Petenyi, 1864).

Specimen IPAE, no. 727/2263 is distinguished from the extant medium-sized members of the genus *Mustela* from Northern Eurasia (*M. erminea* and *M. altaica*) and fossil *M. palerminea* by larger dimensions. In addition, specimen IPAE, no. 727/2263 is distinguished from the above species by the following morphological traits: (1) the relief edge of the temporalis muscle attachment area does not go beyond the posterior margin of m2 in its anterior part and reaches the level of the articular process ventrally; (2) the articular process is positioned lower than the level of the alveolar margin of the mandible; (3) the masseteric fossa extends anteriorly beyond the level of the posterior margin of m1; (4) p4 is large and massive; (5) the anterior cingulid is almost totally absent in p4; (6) the main cusp has a well-developed distal crest in p4; (7) the apex of the main cusp of p4 is higher than the paraconid and protoconid apices of m1; (8) there is no enamel crenulation in p4 and m1; (9) m2 is displaced lingually relative to the m1 axis (occlusal view); and (10) m2 is rather large relative to the m1 dimensions. The trait numbers (with additions) are used below in comparisons with other species.

Specimen IPAE, no. 727/2263 is distinguished from *M. sibirica* by traits (1), (2), and (8); (11) lack of the lingual basin on the m1 talonid; (12) lack of an additional root in the middle part of m1; and (13) lack of a lingual projection (bulge) in the middle part of the m1 crown.

Specimen IPAE, no. 727/2263 is distinguished from *M. putorius* and *M. eversmanii* by traits (1), (2), (6), (8), and (12); (14) a nearly complete lingual cingulid, which is interrupted in the middle part of m1; (15) lack of the hypoconulid in m1; and (16) lingual displacement of m2 relative to m1.

Specimen IPAE, no. 727/2263 is distinguished from *M. lutreola* by traits (1), (2), (8), (11), (13), and (14); and (17) the shearing m1 talonid formed of the hypoconid.

Specimen IPAE, no. 727/2263 is distinguished from *Neogale vison* by traits (8), (13), (14), (16), and (17); (18) lack of cusps in the posterior cingulid of p4; and (19) lack of the buccal cingulid in m1.

Thus, traits (1) and (2) (the relief and position of the edge of the attachment area of the medial part of the temporalis muscle and the position of the articular process) observed in specimen IPAE, no. 727/2263 are not found in the other species of the genus *Mustela*. Kormos [5] has already indicated that the trait of the relief edge of the attachment area of the medial part of the temporalis muscle going beyond the level of the posterior margin of m2 is a species-specific trait of *M. strandi.*

Wiszniowska [6] has considered *M. strandi* to be close to the extant Siberian weasel *M. sibirica*. We similarly observed that the lowest number of traits distinguishes the two species as compared with the other extant species of the genus *Mustela*.

The morphological traits of the dentary and teeth in specimen IPAE, no. 727/2263 are similar to traits described for *M. strandi* [5, 6, 13, 15]. In dimensions, specimen IPAE, no. 727/2263 is closer to the finds from Brasso and Kozi Grzbiet. However, the form from the Taurida cave is characterized by especially large dimensions of the mandible, p4, and m1 and a relatively small m2. It is possible to assume that a trend to a smaller size developed in *M. strandi* in the first half of the Pleistocene. The assumption explains the relatively small sizes of the more recent representatives of the species found in Europe.
