# Erratum to: ***Mustela strandi*** **(Mustelidae, Carnivora) from the Early Pleistocene of Crimea**

**DOI:** 10.1134/S0012496623050010

**Published:** 2024-01-25

**Authors:** D. O. Gimranov, Q. Jiangzuo, A. V. Lavrov, A. V. Lopatin

**Affiliations:** 1 Institute of Plant and Animal Ecology, Ural Branch, Russian Academy of Sciences, 620144 Yekaterinburg, Russia; 2 Key Laboratory of Orogenic Belts and Crustal Evolution, School of Earth and Space Sciences, Peking University, 100871 Beijing, China; 3 Key Laboratory of Vertebrate Evolution and Human Origins of Chinese Academy of Sciences, Institute of Vertebrate Paleontology and Paleoanthropology, Chinese Academy of Sciences, 100044 Beijing, China; 4 Borissiak Paleontological Institute, Russian Academy of Sciences, 117647 Moscow, Russia

The article “*Mustela strandi* (Mustelidae, Carni-vora) from the Early Pleistocene of Crimea,” written by D.O. Gimranov, Q. Jiangzuo, A.V. Lavrov, and A.V. Lopatin, was originally published electronically in Springer-Link on September 12, 2023 without Open Access. After publication in volume 511, pages 284–288, the authors decided to make the article an Open Access publication. Therefore, the copyright of the article has been changed to © The Author(s) 2023 and the article is forthwith distributed under the terms of a Creative Commons Attribution 4.0 International License (http://creativecommons.org/licenses/by/4.0/, CC BY), which permits use, duplication, adaptation, distribution and reproduction of a work in any medium or format, as long as you cite the original author(s) and publication source, provide a link to the Creative Commons license, and indicate if changes were made.

